# Treatment of Immunocompromised, Critically Ill Patients with Influenza A H1N1 Infection with a Combination of Oseltamivir, Amantadine, and Zanamivir

**DOI:** 10.1155/2015/504975

**Published:** 2015-08-09

**Authors:** Wouter J. Meijer, Wiete Kromdijk, Marcel P. H. van den Broek, Pieter-Jan A. Haas, Monique C. Minnema, Charles A. Boucher, Dylan W. de Lange, Annemarie M. J. Wensing

**Affiliations:** ^1^Wilhelmina Children's Hospital, University Medical Center Utrecht, Lundlaan 6, P.O. Box 85090, Utrecht, Netherlands; ^2^Department of Pharmacy & Pharmacology, Slotervaart Hospital, 1066 EC Amsterdam, Netherlands; ^3^Department of Clinical Pharmacy, University Medical Center Utrecht, 3584 CX Utrecht, Netherlands; ^4^Department of Medical Microbiology, University Medical Center Utrecht, 3584 CX Utrecht, Netherlands; ^5^Department of Hematology, University Medical Center Utrecht, 3584 CX Utrecht, Netherlands; ^6^Department of Virology, Erasmus Medical Center, 3015 CE Rotterdam, Netherlands; ^7^Department of Intensive Care, University Medical Center Utrecht, 3584 CX Utrecht, Netherlands; ^8^Department of Virology, Medical Microbiology, University Medical Center Utrecht, 3584 CX Utrecht, Netherlands

## Abstract

Immunocompromised patients are at increased risk of complications of influenza virus infection. We report on two critically ill patients on immunosuppressive medication with influenza pneumonia. In both patients, oseltamivir monotherapy did not result in clearance of the virus after 18 and five days, respectively. After adding zanamivir and amantadine to the treatment, PCRs on pharyngeal and/or plasma specimens turned negative in both patients after four and three days, respectively. We suggest, that in critically ill patients with influenza A H1N1 infection, treatment efficacy should be monitored closely and treatment with a combination of antiviral drugs should be considered.

## 1. Introduction

Influenza virus infection is very common and although usually self-limiting, it remains a significant cause of morbidity in specific vulnerable populations like immunocompromised patients [1, 2]. Treatment with neuraminidase inhibitors is recommended, but viral mutations that reduce susceptibility to oseltamivir occur more often in this group of patients [3, 4]. We report our experience with a combination therapy consisting of oseltamivir, zanamivir, and amantadine.

## 2. Case 1

**Figure 1:**
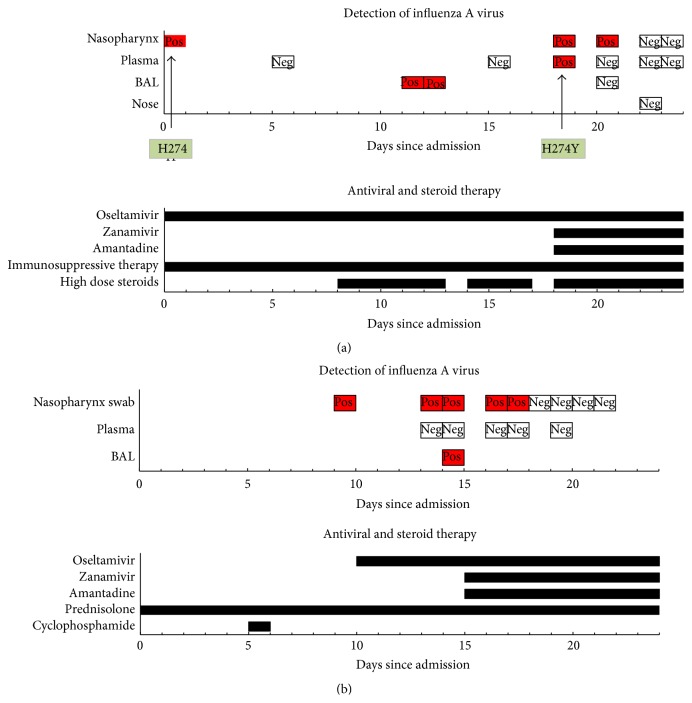
(a) Overview of antiviral and steroid therapy and test results in patient 1. (b) Overview of antiviral and steroid therapy and test results in patient 2.

A 23-year-old woman was admitted to the University Medical Center Utrecht with bilateral pneumonia caused by seasonal influenza A H1N1 virus infection. She was on immunosuppressive medication (prednisolone (20 mg four times daily), mycophenolic acid (2400 mg three times daily), and cyclosporine (100 mg twice daily)) because she had had a myeloablative allogeneic stem cell transplantation as treatment for a relapse of acute myeloid leukemia. Treatment with oseltamivir 75 mg twice daily was initiated, but the condition of the patient worsened and she had to be intubated and was mechanically ventilated. At day 11 a bronchoalveolar lavage was performed and revealed large amounts of influenza A virus (cycle threshold (CT) value 22) despite 10 days of oseltamivir treatment and adequate levels of oseltamivir carboxylate in the serum (Table 1). At day 18 pharyngeal swabs and plasma samples showed H1N1 influenza A virus with a H274Y amino acid substitution in the gene coding for neuraminidase, whereas on admission wildtype H274H had been present. Treatment with zanamivir (10 mg twice daily by nebulization) and amantadine (100 mg twice daily) was added to oseltamivir (Figure 1). Two days after initiation of this combination therapy, influenza virus could no longer be detected in plasma and four days later the pharyngeal specimen turned PCR negative. Nevertheless, the condition of the patient did not improve and she died due to the complication of a tension pneumothorax. Autopsy was not performed.

## 3. Case 2

A 47-year-old man with known polymyositis and scleroderma, but without current immunosuppressive medication, was admitted to our pulmonary unit because of progressive shortness of breath for 4 days. Pneumonia (material obtained from a bronchoalveolar lavage revealed a* Serratia marcescens* infection) and alveolitis were diagnosed and the patient was treated with antibiotics and prednisolone 100 mg/24 h intravenously. On day six, when clinical signs of infection were no longer present, treatment for alveolitis was intensified by adding a dose of 1000 mg of cyclophosphamide. The next day, the patient became more dyspnoeic and had fever. Chest radiography showed progression of the infiltrate. A pharyngeal specimen tested positive for seasonal influenza A H1N1 (CT 33) and treatment with oseltamivir 75 mg twice daily was initiated. Multiple plasma samples tested by PCR were negative for influenza. Three days later, material obtained from a bronchoalveolar lavage and a pharyngeal specimen were still positive for seasonal influenza A H1N1 (CT 32). Subsequently, zanamivir 5 mg twice daily by nebulization (increased the next day to 10 mg twice daily) and amantadine 100 mg twice daily were added to the treatment (Figure 1). No resistance to oseltamivir was identified by genotypic sequence analysis. Four days after initiation of the triple combination therapy, PCR on a pharyngeal swab was negative for influenza virus. Despite this apparent successful treatment, the patient died due to cardiac failure. Postmortem biopsies were taken and tested by PCR for the presence of viral RNA. Tissue samples from liver, spleen, pancreas, bone marrow, heart, both kidneys, and left lung tested negative. However, PCR on tissue from the right lung tested weakly positive for seasonal influenza A H1N1 (CT 39), which suggests incomplete (pulmonary) eradication of the virus. In addition, oseltamivir concentrations in lung and kidney tissue were determined (Table 2).

## 4. Discussion

Neuraminidase inhibitors (oseltamivir, zanamivir, or peramivir) are recommended to treat influenza virus infection [5]. In the past, M2 ion channel inhibitors (such as amantadine) were used as well, but 99% of currently circulating strains are resistant to this class of antiviral drugs. Resistance to neuraminidase inhibitors may also occur, especially after prolonged treatment in immunocompromised patients [4, 6].

Such acquired resistance to oseltamivir was observed in our first patient, where a mutation from wildtype H274H to H274Y in the viral neuraminidase gene occurred during treatment. Since this mutation does not affect the efficacy of treatment with zanamivir and circulating strains were mostly susceptible to amantadine at that time [7] both these drugs were added to the treatment. Meanwhile oseltamivir was maintained, hypothesizing that the continuous selective pressure would result in persistence of the H274Y mutation, which theoretically may impose a barrier for development of resistance to the other antiviral drugs. Shortly after initiation of combination therapy, genomic influenza RNA disappeared both from the plasma and from the pharyngeal specimens.

In our second patient, influenza was only cleared from pharyngeal specimens after initiation of the combination therapy as well. Since both patients were still on immunosuppressive medication and adequate serum levels of oseltamivir carboxylate were achieved before initiation of triple combination therapy (Table 1), we conclude that the antiviral effect is due to the use of this combination therapy.

Although PCR on (pharyngeal and/or plasma) specimens turned negative in both patients, they died due to severe and irreversible organ failure caused by the influenza infection. Detailed postmortem analysis in our second patient revealed a positive influenza PCR on a biopsy of the lung. Although it can be questioned whether the low amount of viral RNA was derived from replication competent virus, these results may indicate incomplete eradication of the virus. Inadequate local levels of the antiviral drugs could cause incomplete eradication. Therefore we determined oseltamivir (carboxylate) concentrations in lung tissue. Considering that in animal models the drug level appears to be higher in lung tissue than in plasma [8], local oseltamivir (carboxylate) levels in our patient seem to be low. Disrupted penetration of the drug due to the (preexistent) destruction of lung tissue or the severe sepsis may have caused these low concentrations, although redistribution of the drug after death cannot be excluded either. Among other factors, the low concentrations may have contributed to the persistent presence of influenza virus RNA. Unfortunately, we were not able to measure levels of the other antiviral drugs amantadine and zanamivir.

Combining different antiviral drugs in the treatment of influenza virus infection has been reported before. A combination of amantadine, oseltamivir, and ribavirin in a group of six relatively mildly affected, immunocompromised patients with influenza A virus infection led to viral clearance in five of these patients [9]. As well, Kim et al. reported a trend towards better survival in critically ill patients with influenza A virus infection treated with the same combination of antiviral drugs [10]. This is in line with studies in mice that have shown that this combination therapy is highly efficacious [11]. Furthermore, the chance of inducing resistance of the virus appears to be reduced when using combination therapy compared to monotherapy [12].

However, treatment with a combination of oseltamivir and zanamivir, whether initiated at once or after initial monotherapy with oseltamivir, resulted in only limited virologic response in critically ill patients [13, 14]. Therefore, questions remain about the best regimen and timing of the initiation of (a combination of) antiviral drugs in the treatment of critically ill patients with influenza A virus infection.

In spite of these considerations, PCR on pharyngeal specimens of both patients turned negative after initiation of combination therapy, indicating suppression of viral replication and shedding. In particular in immunocompromised patients where clearance of the virus by the immune system is limited, using different classes of antiviral drugs that act synergistically could be valuable to suppress viral replication and enhance viral clearance [15]. We suggest that influenza A infection treatment should be monitored closely in these patients, including testing for resistance to antiviral drugs and considering the use of a combination of antiviral drugs. However, further studies on the treatment of immunocompromised, critically ill patients with influenza virus infection are highly necessary.

## Figures and Tables

**Table 1:** Oseltamivir (carboxylate) plasma concentrations expressed as means (range). Concentrations were determined by a validated liquid chromatography tandem mass spectrometry assay.

	Oseltamivir (ng/mL)	Oseltamivir carboxylate (ng/mL)
Patient 1	8.6 (<LLOQ^*∗*^–18,1)	320 (146–614)
Patient 2	2.7 (<LLOQ^*∗*^–3,9)	537 (524–551)

^*∗*^<LLOQ = less than the lower limit of quantification (3 ng/mL for oseltamivir and 10 ng/mL for oseltamivir carboxylate).

**Table 2:** Concentrations of oseltamivir (carboxylate) in lung and kidney tissue of the patient in case two. Samples were obtained postmortem and determined using liquid chromatography/tandem mass spectrometry.

	Oseltamivir (ng/mg tissue)	Oseltamivir carboxylate (ng/mg tissue)
Left lung	<LLOQ	0.19
Right lung	<LLOQ	0.155
Left kidney	0.06	4.53
Right kidney	0.04	3.69

^*∗*^<LLOQ = less than the lower limit of quantification (= 0.015 ng/mg for oseltamivir).
